# An order approach to SPDEs with antimonotone terms

**DOI:** 10.1007/s40072-019-00161-7

**Published:** 2020-01-03

**Authors:** Luca Scarpa, Ulisse Stefanelli

**Affiliations:** 1 Faculty of Mathematics, University of Vienna, Oskar-Morgenstern-Platz 1, 1090 Wien, Austria; 2 Istituto di Matematica Applicata e Tecnologie Informatiche E. Magenes, v. Ferrata 1, 27100 Pavia, Italy

**Keywords:** Existence, Parabolic SPDEs, Antimonotone term, Comparison principle, Order methods, 35K55, 35R60, 60H15

## Abstract

We consider a class of parabolic stochastic partial differential equations featuring an antimonotone nonlinearity. The existence of unique maximal and minimal variational solutions is proved via a fixed-point argument for nondecreasing mappings in ordered spaces. This relies on the validity of a comparison principle.

## Introduction

This note is concerned with the existence of solutions to a class of parabolic stochastic partial differential equations (SPDEs).

The typical setting that we have in mind is the equation

1.1 $$\begin{aligned} \mathrm{d} u - \mathrm{div} (a(\nabla u)) \, \mathrm{d} t - b (u) \, \mathrm{d} t = f(u) \, \mathrm{d} t + G(u) \, \mathrm{d} W\quad \text {in } (0,T)\times \mathcal {O}\end{aligned}$$

suitably coupled with boundary and initial conditions, with $\mathcal {O}$ being a smooth bounded domain of $\mathbb {R}^d$ and $T>0$ a fixed final time.

Here, the real-valued variable *u* is defined on $\Omega \times [0,T]\times \mathcal {O}$, *a* is monotone and polynomial, *f* is Lipschitz continuous, and *G* is a Lipschitz-type operator, stochastically integrable with respect to *W*, a cylindrical Wiener process on the underlying probability space $(\Omega ,\mathscr {F}, \mathbb {P})$. The function $b:\mathbb {R}\rightarrow \mathbb {R}$ is nondecreasing, possibly being nonsmooth, so that the corresponding term in the left-hand side of the equation is indeed antimonotone.

Our aim is to prove that a variational formulation of relation (1.1) admits a solution, whenever complemented with suitable initial and boundary conditions. If *b* is Lipschitz continuous or $-b$ is nondecreasing and continuous such existence follows from the classical theory by Pardoux [13] and Krylov-Rozovskiĭ [8], see also Liu-Röckner [11]. By contrast, we focus here in the case of *b* linearly bounded but not continuous nor nondecreasing.

This situation, to the best of our knowledge, has yet to be addressed. Indeed, the possible discontinuity of $-\,b$ prevents it from being even locally Lipschitz-continuous, hence also the refined well-posedness results for SPDEs with locally monotone or locally Lipschitz-continuous drift (see again [11]) cannot be applied.

The case of a nondecreasing but not Lipschitz continuous nonlinearity *b* in (1.1) prevents from proving existence by a standard regularization or approximation approach. In fact, the usual parabolic compactness seem to be of little use in order to pass to the limit in the antimonotone term $-\,b (u)$. We resort here in tackling the problem in an ordered-space framework instead, by exploiting the fact that *b* is nondecreasing.

At first, we check the validity of a comparison principle by extending to the nonlinear frame of relation (1.1) the corresponding result by Chekroun et al. [6], see Proposition 2.2. This comparison principle allows us to reformulate the existence issue as a fixed-point problem for nondecreasing mappings in ordered spaces. By implementing this fixed-point procedure, we check in Theorem 2.3 that Eq. (1.1) admits variational solutions.

The variational solutions that we obtain via such order method are considered in a *strong* probabilistic sense, i.e. not changing the original stochastic basis and Wiener process. Let us stress that this is extremely satisfactory especially because no uniqueness is to be expected for the Eq. (1.1). Consequently, if one tackled the problem through classical approximation procedures and passage to the limit by stochastic compactness arguments, the nonuniqueness of the limit problem would prevent from obtaining probabilistically strong solutions by the classical procedure à la Yamada–Watanabe [15]. The order argument that we employ is thus efficient in passing by this problem and providing solutions in a strong probabilistic sense even if no uniqueness is expected. Still, one can prove that the set of solutions admits unique maximal and minimal elements in the sense of the pointwise almost-everywhere order.

Before going on, let us mention that order methods for proving existence for SPDEs have already been used in the frame of viscosity solvability. The reader is referred to the seminal papers by Lions and Souganidis [9, 10] as well to [3, 4] for a collection of results in this direction. The novelty here is that we focus on weak solutions instead and that comparison is combined with a fixed-point procedure. The fixed-point Lemma 4.1 corresponds indeed to an abstract version of Perron’s method.

The setting of the problem is discussed in Sect. 2 where we collect some preliminaries and we state our main results, namely Proposition 2.2 (comparison principle) and Theorem 2.3 (existence). The corresponding proofs are then given in Sects. 3 and 4, respectively.

## Setting and main results

The aim of this section is to specify assumptions and introduce a variational formulation for Eq. (1.1), by possibly allowing for additional dependencies in the nonlinearities. Eventually, our main results Proposition 2.2 and Theorem 2.3 are also presented.

Let $(\Omega , \mathscr {F}, (\mathscr {F}_t)_{t\in [0,T]}, \mathbb {P})$ be a complete filtered probability space, where $T>0$ is a given final time, *W* be a cylindrical Wiener process on a separable Hilbert space *U*, and fix a complete orthonormal system $(e_k)_{k\in \mathbb {N}}$ of *U*. The *progressive* $\sigma$-algebra on $\Omega \times [0,T]$ is denoted by $\mathcal P$. For any Banach space *E* and $r,s\in \mathopen [1,\infty \mathclose )$ we denote by $L^r(\Omega ; E)$ and $L^r(0,T; E)$ the usual functional spaces of Bochner *r*-integrable functions and by $L^r_{\mathcal P}(\Omega ; L^s(0,T; E))$ the space of progressively measurable processes $\varphi :\Omega \times [0,T]\rightarrow E$ such that

$$\begin{aligned} \mathop {{}\mathbb {E}}\left( \int _0^T\left\| \varphi (t)\right\| _E^s\,\mathrm{d} t\right) ^{r/s}<\infty . \end{aligned}$$

For any pair of separable Hilbert spaces $E_1$ and $E_2$, the symbol $\mathscr {L}^2(E_1,E_2)$ denotes the space of Hilbert–Schmidt operators from $E_1$ to $E_2$.

Let $\mathcal {O}\subset \mathbb {R}^d$ be nonempty, open, bounded set with Lipschitz boundary. We define the separable Hilbert space

1. $$H:=L^2(\mathcal {O})$$,

and endow it with its usual scalar product $(\cdot ,\cdot )_H$ and norm $\left\| \cdot \right\| _H$. Moreover, we ask

(S2) *V* to be a separable reflexive Banach space, continuously and densely embedded in *H*, that *V* and $V^*$ are uniformly convex and that $V\hookrightarrow L^4(\mathcal {O})$ continuously.

Throughout the paper, we identify *H* with its dual $H^*$ through its Riesz isomorphism, so that the inclusions

$$\begin{aligned} V \hookrightarrow H \hookrightarrow V^* \end{aligned}$$

are continuous and dense. The norm in *V* and the duality between $V^*$ and *V* will be denoted by $\left\| \cdot \right\| _V$ and $\left<\cdot ,\cdot \right>_V$, respectively.

Assumption (S2) is fulfilled for each closed subspace of $W^{1,p}(\mathcal {O})$ for $p \ge 4d /(4+d)$. In particular, homogeneous Dirichlet boundary conditions could be complemented to (1.1) by letting $u\in V=W^{1,p}_0(\mathcal {O})$, other choices being obviously possible. The requirement on *V* and $V^*$ being uniformly convex relates to the validity of a suitable Itô’s formula, [13, Thm. 4.1–4.2].

By allowing additional dependencies, we let the nonlinear function $a: \Omega \times [0,T]\times \mathbb {R}^d \rightarrow \mathbb {R}^d$ in (1.1) possibly depend on time and realization as well. In particular, we ask *a* to be a Carathéodory function, monotone and with *p*-growth with respect to the last variable. This allows to define the operator $A:\Omega \times [0,T]\times V \rightarrow V^*$ as

$$\begin{aligned} \langle Au, v \rangle _V : = \int _\mathcal {O}a(\omega ,t,\nabla u ) \cdot \nabla v \, \mathrm{d} x \quad \forall u,\, v \in V. \end{aligned}$$

By referring now directly to such operator, we assume the following

1. $$A:\Omega \times [0,T]\times V\rightarrow V^*$$ is $$\mathcal P\otimes \mathscr {B}(V)$$–$$\mathscr {B}(V^*)$$ measurable;
2. for every $(\omega ,t)\in \Omega {\times }[0,T]$ and $\varphi ,\psi ,\zeta \in V$ the map $r{\mapsto }\left<A(\omega ,t,\varphi {+} r\psi ),\zeta \right>_V$, $r\in \mathbb {R}$, is continuous;
3. there exist constants $c_A>0$ and $p\ge 2$ such that
  $$\begin{aligned}&\left<A(\omega ,t,\varphi ),\varphi \right>_V\ge c_A\left\| \varphi \right\| _V^p, \end{aligned}$$
  for every $(\omega ,t)\in \Omega \times [0,T]$ and $\varphi \in V$;
4. there exists a constant $C_A>0$ and a progressively measurable process $h\in L^{1}(\Omega \times (0,T))$ such that, setting $q:={p}/{(p-1)}$,
  $$\begin{aligned} \left\| A(\omega ,t,\varphi )\right\| ^q_{V^*}\le C_A\left\| \varphi \right\| _V^{p} + h(\omega ,t) \end{aligned}$$
  for every $(\omega ,t)\in \Omega \times [0,T]$ and $\varphi \in V$;
5. for every increasing Lipschitz-continuous function $\sigma \in C^2(\mathbb {R})$ with $\sigma (0)=0$ it holds
  $$\begin{aligned}&\sigma (\varphi ) \in V \quad \forall \,\varphi \in V,\\&\sigma _{|V}:V\rightarrow V \text { is locally bounded},\\&\left<A(\omega ,t,\varphi ) {-} A(\omega ,t,\psi ),\sigma (\varphi {-}\psi )\right>_V{\ge } 0 \qquad \forall \,(\omega ,t)\in \Omega {\times }[0,T], \quad \forall \,\varphi ,\psi \in V. \end{aligned}$$

Note that (A1)–(A5) hold, for instance, with the choice $a(\omega ,t,\xi ) = \alpha (\omega ,t)|\xi |^{p-2}\xi$ with $\alpha$ measurable, bounded, and uniformly positive.

By choosing $\sigma (r)=r$ in condition (A5) one in particular has that *A* is *monotone*. On the other hand, the choice $\sigma (r)=r^+=\max \{r,0\}$ corresponds to the so-called *T-monotonicity* of *A*, see [2, 5]. These two functions, together with some locally regularised version of $r^+$, see (3.4), are actually the only ones used in the analysis. This would give the possibility of weakening assumption (A5), by explicitly referring to these.

Starting from the Carathéodory function $b: \Omega \times [0,T]\times \mathbb {R}\rightarrow \mathbb {R}$, nondecreasing and linearly bounded in the third variable, we define the operator $B :\Omega \times [0,T]\times H \rightarrow H$ as

$$\begin{aligned} B(\omega ,t,u)(x) = b(\omega ,t,u(x)) \quad \text {for a.e.} \ (\omega ,t,x)\in \Omega \times [0,T] \times \mathcal {O}. \end{aligned}$$

In particular, we require *B* to fulfill

1. *B* is $$\mathcal P\otimes \mathscr {B}(H)$$–$$\mathscr {B}(H)$$ measurable;
2. $$u_1, u_2 \in H, \ u_1 \le u_2$$ a.e. $$\Rightarrow $$ $$B(\cdot ,u_1) \le B(\cdot ,u_2)$$ a.e.
3. there exists a constant $C_B>0$ such that
  $$\begin{aligned} |B(\omega ,t,u(x))| \le C_B\left( 1 + | u (x)| \right) \qquad \forall \, u \in H, \ \text {for a.e.} \ (\omega ,t,x)\in \Omega \times (0,T)\times \mathcal {O}. \end{aligned}$$

Note that no continuity is required on *b* nor or *B*.

Again by possibly allowing additional dependencies, we let the operator $F:\Omega \times [0,T]\times H \rightarrow H$ be defined by

$$\begin{aligned} F(\omega ,t,u)(x) = f(\omega ,t,u(x)) \quad \text {for a.e.} \ (\omega ,t,x)\in \Omega \times [0,T] \times \mathcal {O}\end{aligned}$$

where $f: \Omega \times [0,T]\times \mathbb {R}\rightarrow \mathbb {R}$ is a Carathéodory function, Lipschitz continuous with respect to the last variable. Specifically, we directly assume on the operator *F* the following:

1. *F* is $$\mathcal P\otimes \mathscr {B}(H)$$–$$\mathscr {B}(H)$$ measurable;
2. there exists a constant $C_F>0$ such that,
  $$\begin{aligned} \forall \,u_1,\, u_2 \in H: \quad \Vert F(\cdot ,u_1)-F(\cdot ,u_2)\Vert _H\le C_F \Vert u_1 - u_2\Vert _H \quad \text {a.e. in} \ \ \Omega \times (0,T); \end{aligned}$$
3. there exists $\varphi _F\in H$ such that $F(\cdot ,\cdot ,\varphi _F)\in L^2_{\mathcal P}(\Omega ; L^2(0,T; H))$.

Eventually, the operator $G:\Omega \times [0,T]\times H \rightarrow \mathscr {L}^2(U,H)$ is required to satisfy

1. *G* is $$\mathcal P\otimes \mathscr {B}(H)$$–$$\mathscr {B}(\mathscr {L}^2(U,H))$$ measurable;
2. there exists a constant $C_G>0$ such that, for every measurable subset $\bar{\mathcal {O}}\subset \mathcal {O}$,
  $$\begin{aligned} \sum _{k=0}^\infty \int _{\bar{\mathcal {O}}} |G(\omega ,t,\varphi )e_k-G(\omega ,t,\psi )e_k|^2\le C_G^2\int _{\bar{\mathcal {O}}}|\varphi -\psi |^2 \end{aligned}$$
  for every $(\omega ,t)\in \Omega \times [0,T]$ and $\varphi ,\psi \in H$;
3. there exists $\varphi _G\in H$ such that $G(\cdot ,\cdot ,\varphi _G)\in L^2_{\mathcal P}(\Omega ; L^2(0,T; \mathscr {L}^2(U,H)))$.

Assumption (G2) is a generalized Lipschitz-continuity requirement on *G*. It is not difficult to check that it is satisfied when *G* has the form

$$\begin{aligned} G(\omega ,t,\varphi )e_k=g_k(\omega ,t,\varphi ), \quad k\in \mathbb {N}, \end{aligned}$$

where $g_k:\Omega \times [0,T]\times \mathbb {R}\rightarrow \mathbb {R}$, $k\in \mathbb {N}$, is Carathéodory and

$$\begin{aligned} \sum _{k=0}^\infty |g_k(\omega ,t,r)-g_k(\omega ,t,s)|^2\le C_G^2|r-s|^2, \end{aligned}$$

for every $(\omega ,t)\in \Omega \times [0,T]$ and $r,s\in \mathbb {R}$.

Given the above positions, the variational formulation of (some extension to additional dependencies of) Eq. (1.1) along with variationally defined boundary conditions and an initial condition reads

2.1 $$\begin{aligned} {\left\{ \begin{array}{ll} \mathrm{d} u + A(u)\,\mathrm{d} t - B(u)\, \mathrm{d} t= F(u)\,\mathrm{d} t + G(u)\,\mathrm{d} W \quad \text {in} \ V^*, \ \text {a.e.~in} \ \Omega \times (0,T) ,\\ u(0)=u_0, \end{array}\right. } \end{aligned}$$

As the nonlinear term $-B(u)$ is not monotone and not Lipschitz continuous, existence for (2.1) does not follow from the classical theory [8, 11, 13]. In order to state our existence result, let us first recall a classical statement on well-posedness in case $B=0$: this is a consequence of the classical variational theory for stochastic evolution equations (e.g. [13, Thm. 1.1]).

### Lemma 2.1

(Case $B=0$) Assume *(S1)–(S2)*, *(A1)–(A5)*, *(F1)–(F3)*, and *(G1)–(G3)*. For any initial datum $u_0\in L^2(\Omega ,\mathscr {F}_0; H)$ and any $h \in L^2_{\mathcal P}(\Omega ;L^1(0,T;H))$ the Cauchy problem

2.2 $$\begin{aligned} {\left\{ \begin{array}{ll} \mathrm{d} u + A(u)\,\mathrm{d} t = h\,\mathrm{d} t + F(u)\,\mathrm{d} t + G(u)\,\mathrm{d} W \quad \text {in} \ V^*, \ \text {a.e. in} \ \Omega \times (0,T) ,\\ u(0)=u_0\, \end{array}\right. } \end{aligned}$$

admits a unique solution $u \in L^2(\Omega ; C^0([0,T]; H))\cap L^p_{\mathcal P}(\Omega ; L^p(0,T; V))$, in the sense that

$$\begin{aligned} u(t)+ & {} \int _0^t A(s, u(s))\,\mathrm{d} s {=} u_0 {+} \int _0^t h(s)\,\mathrm{d} s {+}\int _0^tF(s,u(s))\,\mathrm{d} s\\&\quad + \int _0^tG(s,u(s))\,\mathrm{d} W(s) \quad \text {in} \ V^* \end{aligned}$$

for every $t\in [0,T]$, $\mathbb {P}$-almost surely.

The crucial tool in our analysis is a comparison principle for solutions to the Cauchy problem (2.2) with respect to the data. We have the following.

### Proposition 2.2

(Comparison principle) Assume *(S1)–(S2)*, *(A1)–(A5)*, *(F1)–(F3)*, and *(G1)–(G3)*. Let

$$\begin{aligned} u_0^1, u_0^2 \in L^2(\Omega , \mathscr {F}_0; H), \qquad h_1,h_2 \in L^2_{\mathcal P}(\Omega ; L^1(0,T; H)), \end{aligned}$$

and let

$$\begin{aligned} u_1, u_2 \in L^2(\Omega ; C^0([0,T]; H))\cap L^p_{\mathcal P}(\Omega ; L^p(0,T; V)) \end{aligned}$$

be the unique solutions to the Cauchy problem (2.2) with respect to data $(u_0^1, h_1)$ and $(u_0^2, h_2)$, respectively. If

$$\begin{aligned} u_0^1 \le u_0^2 \quad \text {a.e.~in } \Omega \times \mathcal {O}, \qquad h_1\le h_2 \quad \text {a.e.~in } \Omega \times (0,T)\times \mathcal {O}, \end{aligned}$$

then

$$\begin{aligned} u_1(t)\le u_2(t) \quad \text {a.e.~in } \Omega \times \mathcal {O}, \quad \forall \,t\in [0,T]. \end{aligned}$$

The proof of the comparison principle is given in Sect. 3 and corresponds to an extension of the former analogous result by Chekroun et al. [6] to the case of a nonlinear operator *A*.

As the functions $r \mapsto \pm C_B(1+|r|)$, $r\in \mathbb {R}$ are Lipschitz-continuous, owing to Lemma 2.1 we can uniquely find

$$\begin{aligned} u_*,u^* \in L^2(\Omega ; C^0([0,T]; H))\cap L^p_{\mathcal P}(\Omega ; L^p(0,T;V)) \end{aligned}$$

solving the Cauchy problems

2.3 $$\begin{aligned}&{\left\{ \begin{array}{ll} \mathrm{d} u_* + A(u_*)\,\mathrm{d} t \\ = - \,C_B(1+|u_*|)\,\mathrm{d} t + F(u_*)\,\mathrm{d} t + G(u_*)\,\mathrm{d} W\quad \text {in} \ V^*, \ \text {a.e. in} \ \Omega \times (0,T),\\ u_*(0)=u_0, \end{array}\right. } \end{aligned}$$

2.4 $$\begin{aligned}&{\left\{ \begin{array}{ll} \mathrm{d} u^* + A(u^*)\,\mathrm{d} t \\ = C_B (1+|u^*|)\,\mathrm{d} t + F(u^*)\,\mathrm{d} t + G(u^*)\,\mathrm{d} W\quad \text {in} \ V^*, \ \text {a.e. in} \ \Omega \times (0,T) ,\\ u_*(0)=u_0, \end{array}\right. } \end{aligned}$$

respectively. Since $-C_B(1+|r|) \le 0 \le C_B(1+|r|)$, an application of Proposition 2.2 ensures that $u_* \le u^*$ almost everywhere.

We can now state our main result on existence of solutions for the Cauchy problem (2.1).

### Theorem 2.3

(Existence) Assume *(S1)–(S2)*, *(A1)–(A5)*, *(B1)–(B3)*, *(F1)–(F3)*, and *(G1)–(G3)*, Then, for any initial datum $u_0\in L^2(\Omega ,\mathscr {F}_0; H)$ the Cauchy problem (2.1) admits a solution $u \in L^2(\Omega ; C^0([0,T]; H))\cap L^p_{\mathcal P}(\Omega ; L^p(0,T; V))$, in the sense that

$$\begin{aligned} u(t)+ & {} \int _0^tA(s, u(s))\,\mathrm{d} s - \int _0^tB(s, u(s))\,\mathrm{d} s = u_0 + \int _0^t F(s,u(s))\,\mathrm{d} s\\&\quad + \int _0^t G(s, u(s))\,\mathrm{d} W(s) \quad \text {in} \ V^* \end{aligned}$$

for every $t\in [0,T]$, $\mathbb {P}$-almost surely. Moreover, one can uniquely find a minimal solution $u_\mathrm{min}$ and a maximal solution $u_\mathrm{max}$ such that every solution *u* fulfils $u_* \le u_\mathrm{min} \le u \le u_\mathrm{max}\le u^*$ a.e.

The proof of Theorem 2.3 is presented in Sect. 4 and relies on a fixed-point procedure for nondecreasing mappings. Note that no uniqueness for the Cauchy problem (2.1) is to be expected. Indeed, the classical counterexample to uniqueness in $\mathbb {R}$ given by the deterministic ODE problem

$$\begin{aligned} u'=(\max \{u,0\})^{1/2}, \quad u_0=0, \end{aligned}$$

is included in the setting of Theorem 2.3. In this case, $u_\mathrm{min}(t)=0$ and $u_\mathrm{max}(t) = t^2/4$ for $t \ge 0$.

Problem (1.1) and Theorem 2.3 allow for some generalizations. The analysis can be extended to include other significant examples of operators *B* besides $B=b(\cdot )$. For instance, one can consider nonlocal operators $B_{nl}:\Omega \times [0,T]\times H\rightarrow H$ in the form

$$\begin{aligned} B_{nl}(\omega ,t,u)(x){:=}\int _\mathcal {O}\rho (\omega ,t,x-y)b(\omega ,t,u(y))\,\mathrm{d} y, \quad u\in H,\quad (\omega ,t,x)\in \Omega {\times }[0,T]{\times }\mathcal {O}, \end{aligned}$$

where $\rho :\Omega \times [0,T]\times \mathbb {R}^d\rightarrow \mathbb {R}$ is a given random and time-dependent convolution kernel and *b* is as before. It is not difficult to check that $B_{nl}$ satisfies (B1) if $\rho (\cdot ,z)$ is $\mathcal P$-measurable for almost every $z\in \mathbb {R}^d$, $b(\cdot ,w)$ is $\mathcal P$-measurable for almost every $w\in \mathbb {R}$, $\rho (\omega ,t,\cdot )$ is $\mathscr {B}(\mathbb {R}^d)$-measurable, and $b(\omega ,t,\cdot )$ is $\mathscr {B}(\mathbb {R})$-measurable for every $(\omega ,t)\in \Omega \times [0,T]$. Moreover, $B_{nl}$ satisfies (B2) if $\rho \ge 0$ almost everywhere and *b* is increasing in its third variable. Finally (B3) holds for $B_{nl}$ if $\rho \in L^1(\Omega \times (0,T)\times \mathbb {R}^d)$ and *b* is linearly bounded in its third variable. Note that *B* is not necessarily Lipschitz-continuous (and actually not even continuous) is its third variable since *b* may be discontinuous in its third variable.

By suitably strengthening assumptions, some more general classes of operators *G* can be considered as well. In fact, the classical existence result in Lemma 2.1 holds also for operators $G:\Omega \times [0,T]\times V\rightarrow \mathscr {L}^2(U,H)$ as well. In our framework, especially in the context of the comparison principle in Proposition 2.2, such more general class can be handled, provided that further compatibility conditions between *A* and *G* are imposed. For example, if *G* is $\mathcal P\otimes \mathscr {B}(V) / \mathscr {B}(\mathscr {L}^2(U,H))$ measurable and there exists $\varphi _G\in V$ such that $G(\cdot , \cdot , \varphi _G)\in L^2_{\mathcal P}(\Omega ; L^2(0,T; \mathscr {L}^2(U,H)))$, then conditions (G1) and (G3) are straightforwardly extended to this class. The modification of assumption (G2) is more delicate: indeed, by simply requiring *G* to be Lipschitz-continuous from *H* to $\mathscr {L}^2(U,H)$ would imply that *G* can be extended to the whole space *H*, and the dependence on *V* would be absent. A relevant setting where we can allow for an operator *G* defined on *V* is the following: $V=H^1(\mathcal {O})$ (or $V=H^1_0(\mathcal {O})$, depending on the choice of boundary conditions), $p=2$ and assume

(A5)’ $A(\omega ,t,\cdot ):V\rightarrow V^*$ is linear for every $(\omega ,t)\in \Omega \times [0,T]$, and
  $$\begin{aligned}&\left<A(\omega ,t,\varphi ) - A(\omega ,t,\psi ),(\varphi -\psi )^+\right>_V\ge c_A\left\| \nabla (\varphi -\psi )^+\right\| _H^2 \\&\qquad \forall \,(\omega ,t)\in \Omega \times [0,T], \quad \forall \,\varphi ,\psi \in V; \end{aligned}$$
(G2)’ $\exists \, \tilde{C}_G\in (0,\sqrt{2c_A})$ such that, $\forall (\omega ,t)\in \Omega \times [0,T]$ and $\forall \bar{\mathcal {O}}\subset {\mathcal {O}}$ measurable, it holds that
  $$\begin{aligned} \sum _{k=0}^\infty \int _{\bar{\mathcal {O}}} |G(\omega ,t,\varphi )e_k-G(\omega ,t,\psi )e_k|^2\le C_G^2\int _{\bar{\mathcal {O}}}|\varphi -\psi |^2 + \tilde{C}_G^2\int _\mathcal {O}|\nabla (\varphi -\psi )|^2 \quad \forall \,\varphi ,\psi \in V. \end{aligned}$$

By additionally assuming (A5)’–(G2)’, the statement of Proposition 2.2 still holds. We give some detail in this direction at the end of Sect. 3.

## Comparison principle: proof of Proposition 2.2

We closely follow here the argument from [6], by adapting it to our nonlinear setting. Under the notation of Proposition 2.2, we introduce the new variable $u:=u_1-u_2$ and define $h:=h_1-h_2$ and $u_0^:=u_0^1-u_0^2$. Then, *u* satisfies the Cauchy problem

$$\begin{aligned} {\left\{ \begin{array}{ll} \mathrm{d} u + (A(u_1) - A(u_2))\,\mathrm{d} t \\ = h\,\mathrm{d} t + (F(u_1)-F(u_2))\,\mathrm{d} t +(G(u_1)-G(u_2))\,\mathrm{d} W \quad \mathrm{in} V^*, \mathrm{a.e. in} \Omega \times (0,T),\\ u(0) = u_0. \end{array}\right. } \end{aligned}$$

Introduce now the operators

$$\begin{aligned}&\tilde{F}:\Omega \times [0,T]\times H \rightarrow H, \\&\tilde{F}(\omega ,t,\varphi ):=F(\omega ,t,\varphi {+} u_2(\omega ,t)) {-} F(\omega ,t, u_2(\omega ,t)), \quad (\omega ,t,\varphi ) \in \Omega \times [0,T]\times H,\\&\tilde{G}:\Omega \times [0,T]\times H \rightarrow \mathscr {L}^2(U,H), \\&\tilde{G}(\omega ,t,\varphi ):=G(\omega ,t,\varphi {+} u_2(\omega ,t)) {-} G(\omega ,t, u_2(\omega ,t)), \quad (\omega ,t,\varphi ) \in \Omega \times [0,T]{\times } H. \end{aligned}$$

Note that $\tilde{F}$ and $\tilde{G}$ still satisfy assumptions (F1)–(F3) and (G1)–(G3), respectively. Additionally, by definition we have

3.1 $$\begin{aligned} \tilde{F}(\cdot ,\cdot ,0)=0, \qquad \tilde{G}(\cdot , \cdot , 0)=0. \end{aligned}$$

With this notation, the Cauchy problem for *u* can be equivalently rewritten as

3.2 $$\begin{aligned} {\left\{ \begin{array}{ll} \mathrm{d} u {+} (A(u_1) {-} A(u_2))\,\mathrm{d} t {=} h\,\mathrm{d} t {+} \tilde{F}(u)\,\mathrm{d} t +\tilde{G}(u)\,\mathrm{d} W \quad \text {in }V^*,\text { a.e. in }(0,T), \ \mathbb {P}\text {-a.s.}\\ u(0) = u_0. \end{array}\right. } \end{aligned}$$

Recall that we have $u_0\le 0$ a.e. in $\Omega \times \mathcal {O}$ and $h\le 0$ a.e. in $\Omega \times (0,T)\times \mathcal {O}$. Along with this notation, the assertion follows by proving that $u(t)\le 0$ a.e. in $\Omega \times \mathcal {O}$ for all $t\in [0,T]$. We check this by showing that

3.3 $$\begin{aligned} (u(t))^+=0 \qquad \text {a.e.~in } \Omega \times \mathcal {O}, \quad \forall \,t\in [0,T]. \end{aligned}$$

In order to prove (3.3), we resort in an approximation of the positive part by means of the sequence $(\sigma _{\varepsilon })_{\varepsilon >0}$, defined in [6, § 2.4] as

3.4 $$\begin{aligned} \sigma _\varepsilon (r):={\left\{ \begin{array}{ll} r \quad &{}\quad \text {if } r>\varepsilon ,\\ \displaystyle \frac{3}{\varepsilon ^4} r^5 - \frac{8}{\varepsilon ^3}r^4 + \frac{6}{\varepsilon ^2}r^3 \quad &{}\quad \text {if } 0<r\le \varepsilon ,\\ 0 \quad &{}\quad \text {if } r<0. \end{array}\right. } \end{aligned}$$

It is not difficult to check that $\sigma _\varepsilon \in C^2(\mathbb {R})$ for every $\varepsilon >0$, and that there exists a constant $M>0$, independent of $\varepsilon$, such that

3.5 $$\begin{aligned} |\sigma _\varepsilon '(r)| + |\sigma _\varepsilon ''(r)| + |\sigma _\varepsilon (r)\sigma _\varepsilon ''(r)| \le M \qquad \forall \,r\in \mathbb {R}, \quad \forall \,\varepsilon >0. \end{aligned}$$

Moreover, $\sigma _\varepsilon \ge 0$ for every $\varepsilon >0$ and $\sigma _\varepsilon (r)\nearrow r^+$ for all $r\in \mathbb {R}$ as $\varepsilon \searrow 0$. Defining now the primitive functions

$$\begin{aligned} \hat{\sigma }_\varepsilon :\mathbb {R}\rightarrow \mathopen [0,\infty ), \qquad \hat{\sigma }_\varepsilon (r):=\int _0^{r}\sigma _\varepsilon (s)\,\mathrm{d} s, \quad r\in \mathbb {R}, \end{aligned}$$

we introduce the functional $\Sigma _\varepsilon : H \rightarrow \mathopen [0,\infty \mathclose )$ as

$$\begin{aligned} \Sigma _\varepsilon (\varphi ):=\int _\mathcal {O}\hat{\sigma }_\varepsilon (\varphi ), \quad \varphi \in H. \end{aligned}$$

We aim now at applying Itô’s formula to $\Sigma _\varepsilon (u)$. This is indeed possible since $\Sigma _\varepsilon$ is Fréchet differentiable in *H*, with derivative given by

$$\begin{aligned} D\Sigma _\varepsilon : H\rightarrow H, \qquad D\Sigma _\varepsilon (\varphi )=\sigma _\varepsilon (\varphi ), \quad \varphi \in H. \end{aligned}$$

Moreover, since $V\hookrightarrow L^4(\mathcal {O})$, it follows that the restriction of $D\Sigma _\varepsilon$ to *V* is Fréchet differentiable in *V* and its derivative is given by

$$\begin{aligned} D^2\Sigma _\varepsilon :V\rightarrow \mathscr {L}(V,H), \qquad D^2\Sigma _\varepsilon (\varphi )w=\sigma _\varepsilon '(\varphi )w, \quad \varphi ,w\in V. \end{aligned}$$

From (A5) we have that the restriction of $D\Sigma _\varepsilon$ to *V* takes values in *V*, and that $D{\Sigma _\varepsilon }_{|V}:V\rightarrow V$ is strongly-weakly continuous. We can hence apply Itô’s formula in the variational setting of [13, Thm. 4.2] and obtain

$$\begin{aligned}&\Sigma _\varepsilon (u(t)) + \int _0^t\left<A(s,u_1(s))-A(s,u_2(s)),\sigma _\varepsilon (u(s))\right>_V\,\mathrm{d} s \\&\qquad =\Sigma _\varepsilon (u_0) + \int _0^t\left( h(s), \sigma _\varepsilon (u(s))\right) _H\,\mathrm{d} s +\int _0^t\left( \tilde{F}(s,u(s)), \sigma _\varepsilon (u(s))\right) _H\,\mathrm{d} s\\&\quad \qquad +\,\int _0^t\left( \sigma _\varepsilon (u(s)), \tilde{G}(s,u(s))\,\mathrm{d} W(s)\right) _H\\&\quad \qquad +\frac{1}{2}\int _0^t\sum _{k=0}^\infty \left( \sigma _\varepsilon '(u(s))\tilde{G}(s,u(s))e_k, \tilde{G}(s,u(s))e_k\right) _H\,\mathrm{d} s \end{aligned}$$

for every $t\in [0,T]$, $\mathbb {P}$-almost surely. Since $u_0\le 0$ almost everywhere we have $\Sigma _\varepsilon (u_0)=0$. Moreover, since $h\le 0$ and $\sigma _\varepsilon (u)\ge 0$ almost everywhere, the second term on the right-hand side is nonpositive. Noting also that the second term on the left-hand side is nonnegative by (A5), by taking expectations we infer that

$$\begin{aligned} \mathop {{}\mathbb {E}}\Sigma _\varepsilon (u(t))&\le \mathop {{}\mathbb {E}}\int _0^t\left( \tilde{F}(s,u(s)), \sigma _\varepsilon (u(s))\right) _H\,\mathrm{d} s\\&\quad +\,\frac{1}{2}\mathop {{}\mathbb {E}}\int _0^t\sum _{k=0}^\infty \left( \sigma _\varepsilon '(u(s))\tilde{G}(s,u(s))e_k, \tilde{G}(s,u(s))e_k\right) _H\,\mathrm{d} s. \end{aligned}$$

Now, by definition of $\sigma _\varepsilon$, the uniform estimates (3.5), assumptions (F3) and (G3), and the Dominated Convergence Theorem, letting $\varepsilon \searrow 0$ we infer that

$$\begin{aligned} \frac{1}{2}\mathop {{}\mathbb {E}}\Vert u^+(t)\Vert _H^2&\le \mathop {{}\mathbb {E}}\int _0^t\left( \tilde{F}(s,u(s)), u^+(s) \right) _H\,\mathrm{d} s +\frac{1}{2}\mathop {{}\mathbb {E}}\int _0^t\sum _{k=0}^\infty \int _{\{u(s)\ge 0\}}|\tilde{G}(s,u(s))e_k|^2\,\mathrm{d} s\\&=\mathop {{}\mathbb {E}}\int _0^t\!\!\int _{\{u(s)\ge 0\}}\left( \tilde{F}(s,u(s)),u^+(s)\right) _H \,\mathrm{d} s +\frac{1}{2}\mathop {{}\mathbb {E}}\int _0^t\sum _{k=0}^\infty \int _{\{u(s)\ge 0\}}|\tilde{G}(s,u(s))e_k|^2\,\mathrm{d} s\, \end{aligned}$$

for all $t\in [0,T]$. By using the Hölder inequality, the Lipschitz-continuity assumptions (F2) and (G2) on $\tilde{F}$ and $\tilde{G}$, together with the fact that $\tilde{F}(\cdot ,0)=\tilde{G}(\cdot , 0)=0$ from (3.1), we deduce that

3.6 $$\begin{aligned} \frac{1}{2}\mathop {{}\mathbb {E}}\Vert u^+(t)\Vert _H^2&\le \left( C_F + \frac{1}{2}C_G^2\right) \int _0^t \mathop {{}\mathbb {E}}\Vert u^+(s)\Vert _H^2\,\mathrm{d} s. \end{aligned}$$

Hence, (3.3) follows from the Gronwall lemma, and Theorem 2.2 is proved.

With specific reference to the discussion at the end of Sect. 2, let us now mention how the proof can be modified in case of an operator $G:\Omega \times [0,T]\times V\rightarrow \mathscr {L}^2(U,H)$ fulfilling (A5)’–(G2)’. Indeed, the same argument above leads to the following version of relation (3.6)

$$\begin{aligned}&\frac{1}{2}\mathop {{}\mathbb {E}}\Vert u^+(t)\Vert _H^2 + c_A\mathop {{}\mathbb {E}}\int _0^t\left\| \nabla u^+(s)\right\| _H^2\,\mathrm{d} s\\&\qquad \le \left( C_F + \frac{1}{2}C_G^2\right) \int _0^t \mathop {{}\mathbb {E}}\Vert u^+(s)\Vert _H^2\,\mathrm{d} s +\frac{\tilde{C}_G^2}{2}\mathop {{}\mathbb {E}}\int _0^t\left\| \nabla u^+(s)\right\| _H^2\,\mathrm{d} s. \end{aligned}$$

Condition $\tilde{C}_G\in (0,\sqrt{2c_A})$ from (G2)’ allows to rearrange terms and conclude for $u^+=0$.

## Existence of solutions: proof of Theorem 2.3

As anticipated, the proof of Theorem (2.3) relies on a fixed-point tool for nondecreasing mappings in ordered sets. Let us start by recalling some basic notion.

Let $$(E, \preceq ) $$ denote a nonempty ordered set and $$ F\subset E$$. We recall that $$ f \in F $$ is a *maximal (minimal) element* of *F* iff, for all $$f'\in F$$, $$ f\preceq f'$$ ($$ f'\preceq f$$, respectively) implies $$f=f'$$ and that *f* is the *maximum (minimum)* of *F* iff $$ f'\preceq f$$ ($$ f\preceq f'$$, respectively) for all $$ f' \in F$$. Moreover, $$ e \in E$$ is an *upper bound (lower bound)* of *F* iff $$ f\preceq e$$ ($$ e\preceq f$$, respectively) for all $$ f \in F$$ and $$ e\in E $$ is the *supremum* or *least upper bound* (*infimum* or *greatest lower bound*) iff *e* is the minimum (maximum) of the set of upper bounds (lower bounds, respectively) of *F*. Eventually, we say that *F* is a *chain* if it is totally ordered and that *F* is an *interval* iff there exist $$ e_*,e^* \in E $$ such that $$ F\equiv \{ e \in E : e_* \preceq e \preceq e^*\}$$. In the latter case we use the notation $$ F=[e_*,e^*]$$. The set $$(E, \preceq ) $$ is said to be *s-inductive (i-inductive)* iff every chain of *E* is bounded above (below, respectively) and $$ (E, \preceq ) $$ is said to be *completely s-inductive (completely i-inductive)* iff every chain of *E* has a supremum (infimum, respectively). Finally $$ (E, \preceq ) $$ is said to be *inductive (completely inductive)* iff it is both s-inductive and i-inductive (completely s-inductive and completely i-inductive, respectively).

Let us choose $E:=L^2_{\mathcal P}(\Omega ; L^2(0,T; H))$ and specify

$$\begin{aligned} v_1\preceq v_2 \quad \text {iff}\quad v_1\le v_2 \quad \text {a.e.~in } \Omega \times (0,T)\times \mathcal {O}, \qquad v_1,v_2\in E. \end{aligned}$$

By fixing a tentative $\tilde{u} \in E$ in the nonlinearity $-B(\tilde{u})$, one recalls assumptions (B1) and (B3) giving $B(\tilde{u})\in L^2_{\mathcal P}(\Omega ;L^2(0,T;H))$. By using Lemma 2.1, one uniquely finds

$$\begin{aligned} u \in L^2(\Omega ; C^0([0,T]; H))\cap L^p_{\mathcal P}(\Omega ; L^p(0,T; V))\subset E \end{aligned}$$

solving the Cauchy problem

$$\begin{aligned} {\left\{ \begin{array}{ll} \mathrm{d} u + A(u)\,\mathrm{d} t = B(\tilde{u})\,\mathrm{d} t + F(u)\,\mathrm{d} t + G(u)\,\mathrm{d} W \quad \text {in} \ V^*, \ \text {a.e. in} \ \Omega \times (0,T) ,\\ u(0)=u_0. \end{array}\right. } \end{aligned}$$

This defines a mapping $S:E \rightarrow E$ as

$$\begin{aligned} S(\tilde{u}):=u. \end{aligned}$$

The function $u \in E$ is hence a solution of the Cauchy problem (2.1) if and only if it is a fixed point of *S*. We will use the following fixed-point lemma.

### Lemma 4.1

(Fixed point) Let $(E, \preceq )$ be an ordered set and $I:= [e_*,e^*] \subset E$ be completely inductive. Suppose that $S: (I,\preceq ) \rightarrow (I,\preceq )\,$ is nondecreasing. Then, the set of fixed points $\{ u \in I \ : \ u = S(u)\}$ is nonempty and has a minimum and a maximum.

This fixed-point result was announced by Kolodner [7] and turns out to be the main tool in the analysis of [12, 14]. Its proof is to be found, for instance, in [1, Thm. 9.26, p. 223]. This fixed-point lemma corresponds indeed to an abstract version of the classical Perron’s method. In particular, in order to identify the unique minimal fixed point of *S* one subsequently proves that the set of *subsolutions* $A:=\{v \in I \ : \ v \preceq S(v) \}$ is non-empty, *A* with the induced order is completely s-inductive, *A* has a maximal element *u*, and *u* is a fixed point for *S*.

In order to apply the fixed-point Lemma 4.1 we define $e_*=u_*$ and $e^*=u^*$, where $u_*$ and $u^*$ are the unique solutions to (2.3) and (2.4), respectively, and check that (1) *I* is completely inductive, (2) *S* is nondecreasing, and (3) $S(I)\subset I$.

1. Let $\emptyset \not = F \subset I$ be a chain. For almost all $(\omega ,t ,x)\in \Omega \times (0,T)\times \mathcal {O}$ we have $(\sup F)(\omega ,t ,x) =\sup \{u (\omega ,t ,x) \ | \ u \in F\}$ and $(\inf F)(\omega ,t ,x) =\inf \{u (\omega ,t ,x) \ | \ u \in F \}$, so that $\sup F,\, \inf F \in I$. Hence, *I* is completely inductive.
2. Take $\tilde{u}_1 \preceq \tilde{u}_2$ and recall that $u_1 = S(\tilde{u}_1)$ and $u_2 = S(\tilde{u}_2)$ are the unique solutions to the Cauchy problem (2.2) with *h* replaced by $h_1=B(\tilde{u}_1)$ and $h_2=B(\tilde{u}_2)$, respectively. As *B* is nondecreasing, we have that $h_1 \preceq h_2$. By applying Proposition 2.2 we then find $u_1 \preceq u_2$. This proves that $S(\tilde{u}_1)\preceq S(\tilde{u}_2)$, namely *S* is nondecreasing.
3. Let $\tilde{u}\in I$ and set $u = S(\tilde{u})$. As $u_*\preceq \tilde{u}$ and *B* is nondecreasing, we have that $B(u_*)\preceq B(\tilde{u})$. Assumption (B2) ensures that we have that
  4.1 $$\begin{aligned} -C_B(1+|v|) \le |B(\cdot ,v)|\le C_B(1+|v|) \quad \forall \,v \in H, \ \text {a.e. in} \ \Omega \times (0,T)\times \mathcal {O}. \end{aligned}$$
  Consequently, we deduce that
  $$\begin{aligned} -C_{B}(1+|u_*|)\le B(\cdot , u_*)\le B(\cdot , \tilde{u}) \quad \text {a.e.~in } \Omega \times (0,T)\times \mathcal {O}. \end{aligned}$$

Noting also that $u_*(0)= \tilde{u} (0)=u_0$, we can apply Proposition 2.2 with the choices $u_0^1=u_0^2=u_0$, $h_1=-C_{B}(1+|u_*|)$, and $h_2= B(\cdot ,\tilde{u})$ and deduce that $u_*\preceq S(\tilde{u})$. An analogous argument entails the upper bound $S(\tilde{u}) \preceq u^*$, so that $u_* \preceq S(\tilde{u}) \preceq u^*$ or, equivalently, $S(\tilde{u})\in I$.

We are hence in the position of applying Lemma 4.1 and find that the set of fixed points of *S* in *I* is nonempty and has (unique) maximum and minimum. The proof of Theorem 2.3 follows then by checking that *all* solutions *u* to the Cauchy problem (2.1) necessarily belong to *I*. This follows by applying once again Proposition 2.2 and using relations (4.1).

The comparison principle from Theorem 2.2 can be used to constructively find a solution to some weaker version of the Cauchy problem (2.1) by an iterative procedure. Indeed, in the setting of Theorem 2.3 one can let $u^0=u_*$ (an analogous argument applies to the choice $u^0=u^*$) and iteratively define a sequence $(u^n)_n$ by letting

4.2 $$\begin{aligned} u^{n+1}=S(u^n), \end{aligned}$$

i.e., by variationally solving

4.3 $$\begin{aligned} \mathrm{d} u^{n+1} {+} A(u^{n+1})\,\mathrm{d} t \,{=}\, B(u^n)\,\mathrm{d} t {+} F(u^{n+1})\,\mathrm{d} t {+} G(u^{n+1})\,\mathrm{d} t, \quad u^{n+1}(0)\,{=}\,u_0, \quad n{\ge }1. \end{aligned}$$

Owing to the fact that *S* is nondecreasing, one readily checks that

$$\begin{aligned} u^0=u_*\preceq S(u_*) = u^1 \Rightarrow u^1=S(u^0) \preceq S(u^1)=u^2 \Rightarrow u^n \preceq u^{n+1}, \end{aligned}$$

so that the sequence $(u^n)_n$ is nondecreasing. Since $u^{n+1} =S(u^n)\preceq u^*$, the Monotone Convergence Theorem ensures that $u^n \rightarrow u$ strongly in $L^2_{\mathcal P}(\Omega ;L^2(0,T;H))$ to some limit *u*. The linear bound on *B* and the classical estimates entail sufficient compactness to pass to limits in (4.3). Most notably, as *B* is not decreasing and linearly bounded, the sequence $B(u_n)$ is also monotonically converging, namely, $B(u_n) \rightarrow \hat{B}$ strongly in $L^2_{\mathcal P}(\Omega ;L^2(0,T;H))$. As $u \mapsto b(\omega , t, u)$ is not required to be continuous, one cannot conclude that $\hat{B} = b(\cdot ,u)$ a.e. Rather, we obtain the weaker identification $\hat{B} \in \overline{b}(\cdot , u)$ a.e., where, for all $(\omega ,t)\in \Omega \times [0,T]$, we have defined

$$\begin{aligned} \overline{b}(\omega ,t,z):=\{ \xi \in \mathbb {R}\ : \ \exists \, z_n \rightarrow u \ \text {in} \ \mathbb {R}, \ b(\omega ,t,z_n) \rightarrow \xi \}, \quad (\omega ,t,z)\in \Omega \times [0,T]\times \mathbb {R}. \end{aligned}$$

Namely, $\overline{b}(\omega ,t,\cdot )$ corresponds to the closure in $\mathbb {R}\times \mathbb {R}$ of the graph of $b(\omega ,t,\cdot )$. In particular, in case *b* is discontinuous in its third argument, the iterative procedure (4.2) does not allow to recover the existence result of Theorem 2.3.
